# Annotations

**Published:** 1893-12-23

**Authors:** 


### ANNOTATIONS.

### The Air of Schools.

An important paper is published in the November number of the *Journal of Pathology* on the "Air of Schools." The paper has been prepared for publication by Mr. J. Foggie, F.C.S., from materials accumulated chiefly by the late Professor Carnelley, of the University of Aberdeen. Some interesting and practically important results may be briefly indicated. Professor Carnelley found, among other things, that the air of town schools is less pure than that of country schools, and that purity increases in direct proportion to the distance from town. That, of course, will surprise no one. He also found that in proportion as schools and their pupils were "clean," "medium," or "dirty," so was the number of injurious micro-organisms in the air of the examined school-rooms, small, larger, or largest. For example, in 36 schoolrooms which were classed as "clean" there was found an average of 85 micro-organisms to every litre of air. In 39 classed as "medium" there were 94 to every litre, whilst in 34 "dirty" schoolrooms there were as many as 139 micro-organisms to every litre of air. A similar, but much more marked, disproportion in the number of micro-organisms was found when the children, rather than the schools, were the subjects of observation. Thus in the case of 32 schools where the children were "clean" there were found 63 micro-organisms in every litre of air; in 46, where they were only "medium," there were 99 micro-organisms to the litre; and in 31, where the children were pronounced "dirty," there were 159 micro-organisms to every litre of atmospheric air, or two and a half times as many as among the children who were "clean;" from which we infer that dirty persons are much more likely to spread infectious diseases than dirty dwellings. The schools examined, it should be noted, were Board schools; and the practical conclusion we wish to draw is this—that the next great reform in our elementary education programme must be twofold; first, the compulsory cleansing and purifying of all school buildings at least twice a year; and, secondly, the compulsory washing and bathing of all school children by the school authorities at least twice a week. If their clothing could also be compulsorily cleansed and disinfected twice a month, so much the better.

### Life on Threepence Three Farthings a Day.

The corporal of cavalry who by patient diminution succeeded in reducing his horse's rations to a straw a day must have been a Devonshire man, and his birthplace was, no doubt, the parish of Newton Abbott. The guardians of that same parish are now devoting themselves to the interesting biological problem of raising pauper boys to British manhood on the smallest possible amount of sustenance compatible with life. The present cost of each inmate in the Newton Workhouse, according to the *Western Mercury*, is 2s. 1d. per week, or something less than 3¾d. per day. On this generous fare the boys above nine years of age have positively declined to put on a rosy appearance, or to show any spirit whatever, such as is appropriate to that age. On the contrary, they appear to look "pale and sickly," and generally to set a bad physiological example to the boyhood of the parish. Under these circumstances, Colonel Walcott has proposed to improve their diet to the extent of 1½d. per week—a little less than a farthing a day. Dr. Ley, himself a physiologist, seconded this innovating motion. But Mr. G. Stooke, who said he had attended guardians' meetings under five different chairmen, had never before heard of the children's daring to look "pale and sickly." Opposed by such weighty authority as this, the motion of Colonel Walcott and Dr. Ley was deferred for a fortnight. Dr. Ley stated that the present dietary was established in 1850, and was the poorest in Devonshire. No doubt, if every other county were polled, it would be found to be also the poorest in England; and probably, if the Newton Guardians were measured, their heads would prove the smallest and their abdominal circumference the biggest to be found in England. Feelings in this case are out of the question. A certain amount of intellect is required before feeling is developed in any sort of animal, human or otherwise. The Newton Guardians were all manifestly born before the passing of Mr. Forster's Elementary Education Act.

### Birds and Big Dogs.

"No subscriptions asked!" This might be the appropriate motto of one of the kindest and most necessary societies we have—the Society for the Protection of Birds. The society is making a little spurt this Christmas; but the "spurt" is not half energetic enough. Something more must be done soon, or all our larks will be "limed" by the lazy bird-catcher, and gobbled up by the greedy gourmand who would eat his grandmother if she were not too tough to be nice. Just now the society is "down upon" the fashionable women who insist upon adorning themselves with feathers and dead birds. Quite right! Let all such women be anathema! But will not the society spare a leaflet for the larks? How those glorious little creatures used to flood the air with song, and fill the pedestrian's heart with gladness as he walked across a certain southern common well known to the writer! But last summer was the finest lark-season known in the memory of man; and yet on that very common there was scarcely a lark to be seen or heard even on the very sunniest of days! How could there be? All the previous winter the lazy vagabonds of bird-catchers had been at their cruel trade! This winter the same thing is happening in the same place. It is no doubt happening all over England. Whilst we plead that the birds may have their fair share of Christmas pudding, and may have all the ripe, red berries left on the trees for their winter use, we plead also that everybody who has a field or a "common right" will keep a big dog, and train him to make bird-catchers "fly" more quickly than the birds. Of all the charms of our English summer landscapes, none are more dear or more truly English than our sweet singing birds. Everybody ought to belong to the Birds Protection Society; and everybody may become a member of it by paying twopence a year for a "card"; whilst all the fuller privileges of associateship can be purchased for a shilling per annum. The hon. secretary of the society is Mrs. F. E. Lemon, Hillcrest, Redhill.

